# Effect of TiO_2_ nanoparticle-accumulated bilayer photoelectrode and condenser lens-assisted solar concentrator on light harvesting in dye-sensitized solar cells

**DOI:** 10.1186/1556-276X-8-283

**Published:** 2013-06-12

**Authors:** Kook Joo Moon, Sun Woo Lee, Yong Hun Lee, Ji Hoon Kim, Ji Young Ahn, Seung Jun Lee, Deug Woo Lee, Soo Hyung Kim

**Affiliations:** 1 Department of Samsung Advanced Integrated Circuit Engineering, Pusan National University, 30 Jangjeon-dong, Geumjung-gu, Busan 609-735, South Korea; 2 Department of Nanomechatronics Engineering, Pusan National University, 30 Jangjeon-dong, Geumjung-gu, Busan 609-735, South Korea; 3 Department of Nano Fusion Technology, Pusan National University, 30 Jangjeon-dong, Geumjung-gu, Busan 609-735, South Korea

**Keywords:** Dye-sensitized solar cell, Solar concentrator, Plano-convex lens, Focal length, Light-scattering layer

## Abstract

TiO_2_ nanoparticles (NPs) with a size of 240 nm (T240), used as a light-scattering layer, were applied on 25-nm-sized TiO_2_ NPs (T25) that were used as a dye-absorbing layer in the photoelectrodes of dye-sensitized solar cells (DSSCs). In addition, the incident light was concentrated via a condenser lens, and the effect of light concentration on the capacity of the light-scattering layer was systematically investigated. At the optimized focal length of the condenser lens, T25/T240 double layer (DL)-based DSSCs with the photoactive area of 0.36 cm^2^ were found to have the short circuit current (*I*_sc_) of 11.92 mA, the open circuit voltage (*V*_oc_) of 0.74 V, and power conversion efficiency (PCE) of approximately 4.11%, which is significantly improved when they were compared to the T25 single layer (SL)-based DSSCs without using a solar concentrator (the corresponding values were the *I*_sc_ of 2.53 mA, the *V*_oc_ of 0.69, and the PCE of 3.57%). Thus, the use of the optimized light harvesting structure in the photoelectrodes of DSSCs in conjunction with light concentration was found to significantly enhance the power output of DSSCs.

## Background

Dye-sensitized solar cells (DSSCs) have been developed extensively because of the relatively low cost involved in their manufacturing processes [1]. Numerous research groups have reported the enhancement of the light-to-electricity power output of DSSCs by employing newly developed materials and modifying the intrinsic solar cell structures [2-10]. An alternative approach for enhancing the light-to-electricity power output of DSSCs is to use a solar concentrator, which generally employs optical lenses or mirrors [11,12]. The optical lens is incorporated to improve the power output of photovoltaic cells (PVs) by concentrating a large amount of sunlight onto a small area of photoactive layers in various PVs. In general, the power output of DSSCs decreases with an increase in the cell area of the photoactive layer. However, this problem can be solved by employing a solar concentrator that provides the advantages of increased power output. The application of an optical lens-based solar concentrator system mounted on top of DSSCs still poses several challenges in terms of efficiency, cost-effectiveness of optical design, and the provision of uniform and concentrated illumination on a DSSC regardless of surrounding environmental changes [13-15]. Furthermore, various complex phenomena, including light scattering, recombination of electron-hole pairs, and dye degradation, in the photoactive layers of DSSCs can occur when the intensity of incident light is changed by varying the beam focus of solar concentrator [16]. The question arises as to how we can optimize the effects of the intrinsic cell structure and solar concentrator when concentrated light is incident on the photoactive layer structures in DSSCs.

In this work, we systematically investigated the effects of using a light-scattering layer in the photoelectrodes of DSSCs along with studying the effects of using a condenser lens-based solar concentrator on the photovoltaic performance of DSSCs. Briefly, three different photoelectrode structures fabricated with a T25/T25-accumulated double layer (T25/T25 DL), a T25/T240-accumulated double layer (T25/T240 DL), and a T240/T240-accumulated double layer (T240/T240 DL) were examined for verifying the effects of using a light-scattering layer under intensified light irradiation conditions tuned by a condenser lens-based solar concentrator. Here, T25 and T240 indicate commercialized TiO_2_ nanoparticles (NPs) with an average diameter of approximately 25 and 240 nm, respectively. With the optimized design of the condenser lens-based solar concentrator developed in this approach, we report a novel T25/T240 DL-based DSSC system with condenser lens-based solar concentrator that exhibits a photocurrent output of approximately 11.92 mA, an open circuit voltage of 0.74 V, and power conversion efficiency (PCE) of approximately 4.11%, which exhibits a much better photovoltaic performance compared to T25/T25 DL- and T240/T240 DL-based DSSCs with condenser lens-based solar concentrator.

## Methods

**Figure 1:**
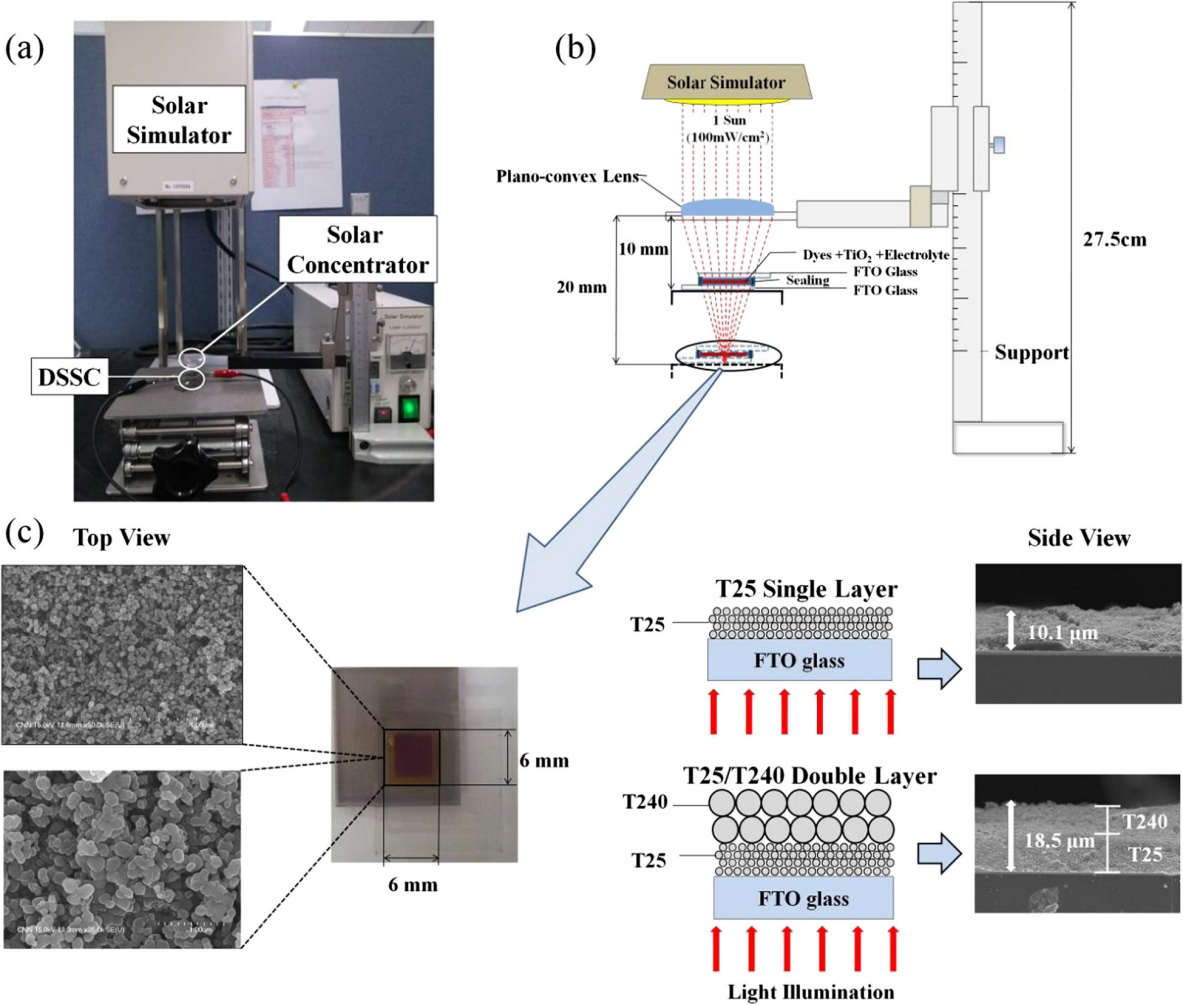
**Experimental setup for measuring the photovoltaic performance of DSSCs.** (**a**) Photograph of the DSSC, condenser lens-based solar concentrator system, and solar simulator, (**b**) schematic of light pathways in condenser lens-based solar concentrator system, and (**c**) SEM images of top view and side view of TiO_2_ NP-accumulated photoelectrode of the DSSC (Here, T25 single layer: 25-nm-sized TiO_2_ NP layer; T25/T240 double layer: 240-nm-sized TiO_2_ NP light-scattering layer applied on 25-nm-sized TiO_2_ NP layer).

Commercially available TiO_2_ NPs (T25, Degussa; T240, Sigma Aldrich, St. Louis, MO, USA) were used without further treatment. In order to prepare TiO_2_ NP paste for the screen-printing process, 6 g of TiO_2_ NPs, 15 g of ethanol, 1 mL of acetic acid (CH_3_COOH), and 20 g of terpineol were mixed in a vial and sonicated for 1 h. A solution of 3 g of ethylcellulose dissolved in 27 g of ethanol was separately prepared and subsequently added to the TiO_2_ NP-dispersed solution, which was then sonicated for 30 min [5,17]. As a photoelectrode layer, TiO_2_ NP-accumulated thin layer was applied via a screen-printing process on a fluorine-doped tin oxide (FTO) glass (SnO_2_:F, 7 Ω/sq, Pilkington, Boston, USA) with a photoactive area of 0.6 × 0.6 cm^2^, as shown in Figure 1. The T25 single layer (T25 SL), T25/T25 DL, T25/T240 DL, and T240/T240 DL were separately prepared for comparison purposes. The resulting TiO_2_ NP-accumulated layer formed on the FTO glass via the screen-printing process was then sintered in an electric furnace at 500°C for 30 min and subsequently immersed in anhydrous ethanol containing 0.3 mM of Ru dye (Bu_4_N)_2_[Ru(Hdcbpy)_2_-(NCS)_2_] (N719 dye, Solaronix, Zollikon, Switzerland) for 24 h at room temperature in order to allow the dye molecules to attach themselves to the entire surface of the TiO_2_ NPs. The dye-soaked TiO_2_-NP-based photoelectrode was then rinsed with ethanol and dried in a convection oven at 80°C for 10 min. As a counter electrode, we prepared Pt-coated FTO glass using an ion sputter (model no. E1010, Hitachi, Chiyoda-ku, Japan) operated at 2.5 kV. Both the dye-soaked TiO_2_ NP-based photoelectrode and the Pt-coated counter electrode were sealed together with a hot-melt polymer film (60-μm thick, Surlyn, DuPont, Wilmington, Delaware, USA) that was inserted between them, and an iodide-based liquid electrolyte (AN-50, Solaronix) was then injected into the interspace between the electrodes. The current-voltage (*I–V*) characteristics of the resulting DSSCs fabricated in this study were measured under AM 1.5 simulated illumination with an intensity of 100 mW/cm^2^ (PEC-L11, Peccell Technologies, Inc., Yokohama, Kanagawa, Japan). The intensity of sunlight illumination was calibrated using a standard Si photodiode detector with a KG-5 filter. The *I–V* curves were automatically recorded using a Keithley SMU 2400 source meter (Cleveland, OH, USA) by illuminating the DSSCs. The condenser lens-based solar concentrator employed in this study had a diameter of 15 mm, a center thickness of 3.35 mm, an edge thickness of 1.36 mm, and an effective focal length of 22.5 mm. The condenser lens was supported by a homemade vertical holder, and the focal length was changed by adjusting the rotating gauge.

## Results and discussion

First, in order to examine the effects of the condenser lens-based solar concentrator on the photovoltaic performance of DSSCs, we varied the focal length of the light pathway in the condenser lens system such that a reference DSSC with an approximately 10-μm-thick T25 single layer (T25 SL) was exposed to various concentrated sunlight conditions, as shown in Figure 1. Here, by simulating the optical geometries in the given condenser lens system, we estimated that the circular area of the focused beam can fully cover a 0.6 × 0.6 cm^2^ photoactive layer as long as the optical length is less than 10 mm. Also, when condenser lens system was applied, the temperature measured by a thermocouple installed on top of DSSC was approximately 40°C or less, in which no additional cooling system was required.

From Table 1, we can clearly observe that the short circuit current (*I*_sc_) significantly increases with the focal length of the condenser lens system (i.e., higher light concentration). With the use of the condenser lens system, the PCE of the reference T25 SL-based DSSC was found to slightly decrease from approximately 3.57% (without the condenser lens) to approximately 3.38%, when the focal length was set to the maximum value of approximately 10 mm. This is owing to the increase of power input caused by higher light concentration with longer focal length. However, as the light concentration increased, both *I*_sc_ and *V*_oc_ were observed to make a significant increase. This is consistent with the general theoretical model given in Equation 1 for conventional inorganic solar cells that *I*_sc_ increases linearly with increasing light intensity (*X*), and *V*_oc_ increases logarithmically with increasing *I*_sc_ and *X*:

$$\;V_{\mathrm{OC}}\approx \frac{\mathrm{nkT}}{q}\ln\left(\frac{X\cdot I_{\mathrm{SC}}}{I_{o}}\right)=\frac{\mathrm{nkT}}{q}\ln\left(X\right)+\mathrm{const}$$

where, *n* is the diode quality factor, *k* is the Boltzmann's constant, *T* is the absolute temperature, *q* is the electronic charge, and *I*_o_ is the reverse saturation current.

**Table 1:** Summary of photovoltaic characteristics of T25-accumulated single layer (T25 SL)-based DSSCs

**Type**	**Condenser lens**	**Focal length (mm)**	**Light concentration (Suns)**	***I***_**sc **_**(mA)**	***V***_**oc **_**(V)**	**FF**	**PCE (%)**
T25 SL	Without	-	1.00	2.53	0.69	0.74	3.57
	With	6	2.12	5.27	0.73	0.69	3.47
		7	2.44	6.01	0.73	0.68	3.41
		8	2.78	6.95	0.73	0.67	3.41
		9	3.24	8.14	0.74	0.66	3.40
		10	3.72	9.35	0.74	0.65	3.38

*I*_sc_, photocurrent; *V*_*oc*_, open circuit voltage; FF, fill factor; PCE, power conversion efficiency.

**Figure 2:**
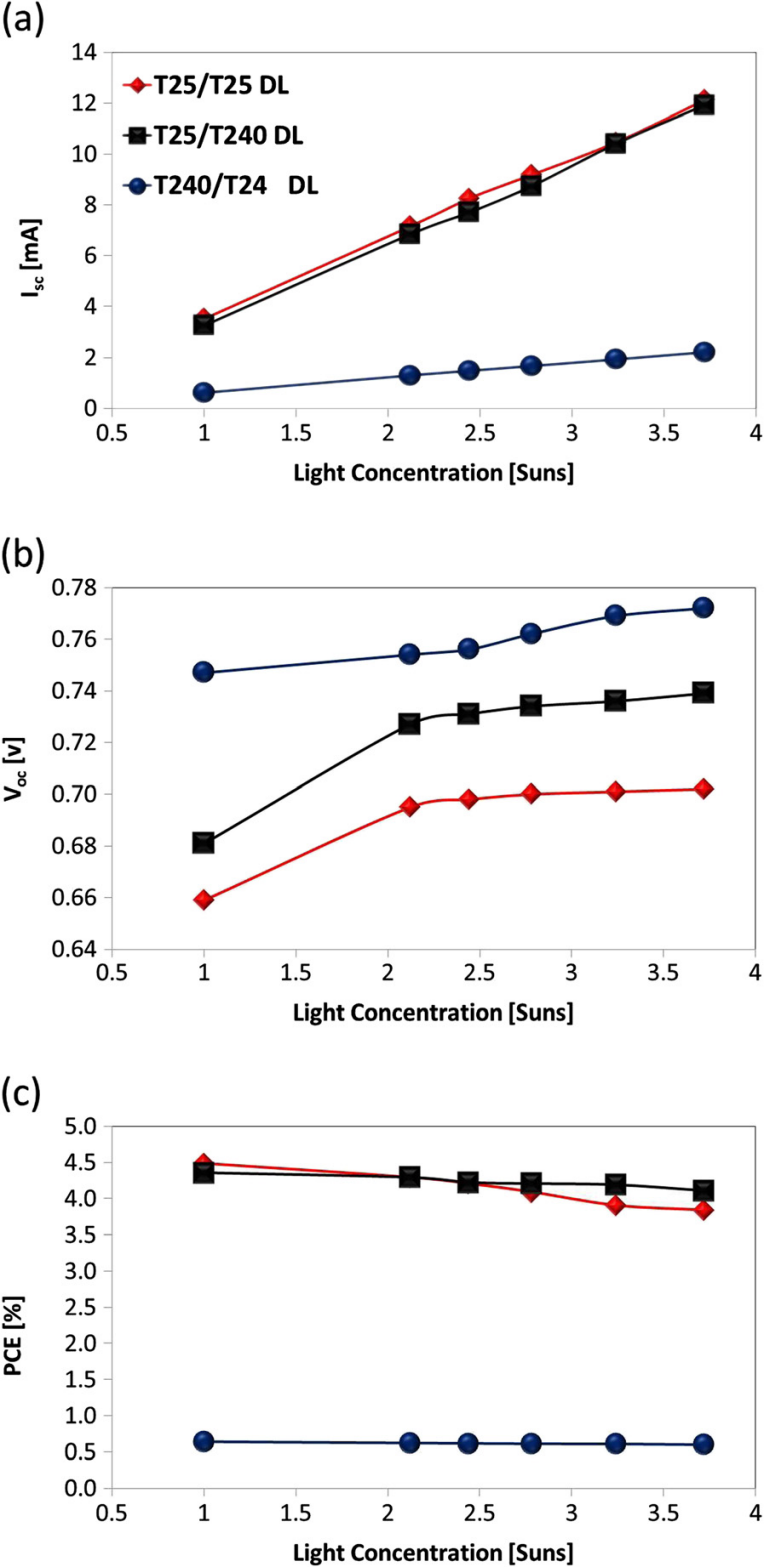
**Photovoltaic properties of T25/T25-DL-, T25/T240-DL-, and T240/T240-DL-based DSSCs.** The evolution of (**a**) *I*_sc_, (**b**) *V*_oc_, and (**c**) PCE of T25/T25-DL-, T25/T240-DL-, and T240/T240-DL-based DSSCs as a function of light concentration.

In order to examine the effect of the TiO_2_ light-scattering layer on the performance of DSSCs, we fabricated three different DSSCs with photoelectrodes composed of (1) a T25/T25 DL, (2) T25/T240 DL, and (3) T240/T240 DL with a total thickness of approximately 18 μm. After the T240-accumulated light-scattering layer was applied on the T25 layer, the resulting PCE of the fabricated DSSCs without condenser lens improved from approximately 3.57% (i.e., T25-SL-based DSSC, Table 1) to approximately 4.36% (i.e., T25/T240-DL-based DSSC, Figure 2c), corresponding to an approximately 22% increment. This suggests that the T240-accumulated layer could play the role of dye molecule absorbing or light scattering or both. The former can be directly ascertained by examining the photovoltaic performance of the DSSC based on a T240/T240-DL-based photoactive layer as shown in Figure 2. Consequently, an *I*_sc_ of 0.62 mA, a *V*_oc_ of 0.75, a fill factor (FF) of 0.50, and a PCE of 0.64% were obtained for the DSSC based on the T240/T240-DL-based photoactive layer under a 1 sun condition at AM 1.5, indicating that the number concentration of photogenerated electrons is negligibly small and the role of the absorbing dye molecules in increasing the PCE in the pure T240-accumulated layer is relatively very weak. Therefore, the higher PCE obtained for the T25/T240-DL-based DSSC when compared with that of the T25-SL-based DSSC is a consequence of greater light scattering. As shown in Figure 2a, the values of *I*_sc_ of both T25/T25 DL and T25/T240 DL were very similar and significantly increased with increasing the light concentration. Also, the presence of larger primary size of TiO_2_ NPs (i.e., T240) in the photoelectrode generated higher value of *V*_oc_ than smaller TiO_2_ NPs (i.e., T25), and the value of *V*_oc_ was increased with increasing the light concentration as shown in Figure 2b. Therefore, the resulting PCE of T25/T240-DL©-based DSSCs remained very stably with the highest values under the higher light concentrations as shown in Figure 2c. Here, © denotes the condenser lens-based solar concentrator installed on top of DSSCs.

**Figure 3:**
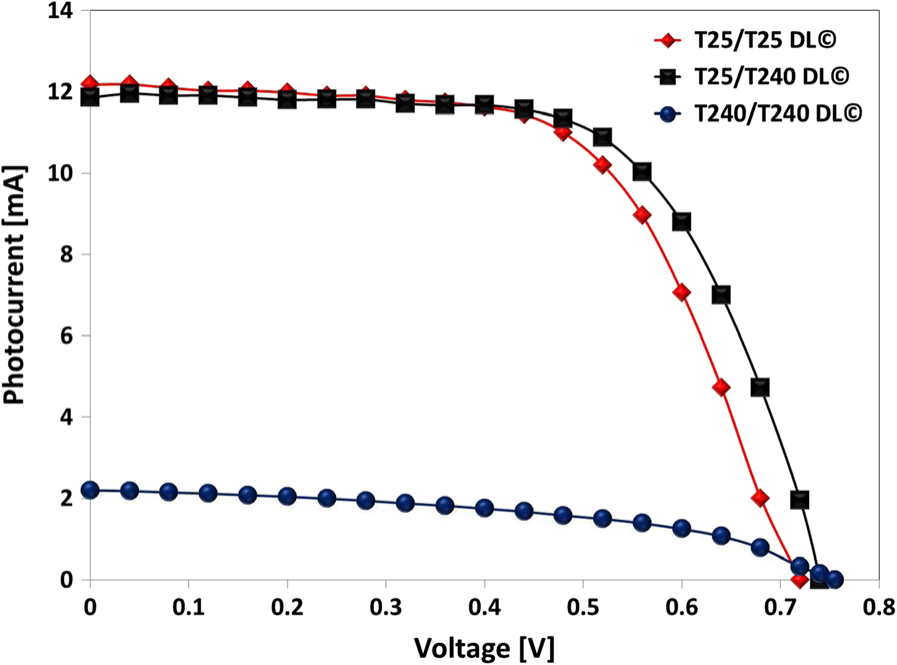
***I*–*V *characteristics of T25/T25-DL-, T25/T240-DL-, and T240/T240-DL-based DSSCs with condenser lens-based solar concentrator.**

**Figure 4:**
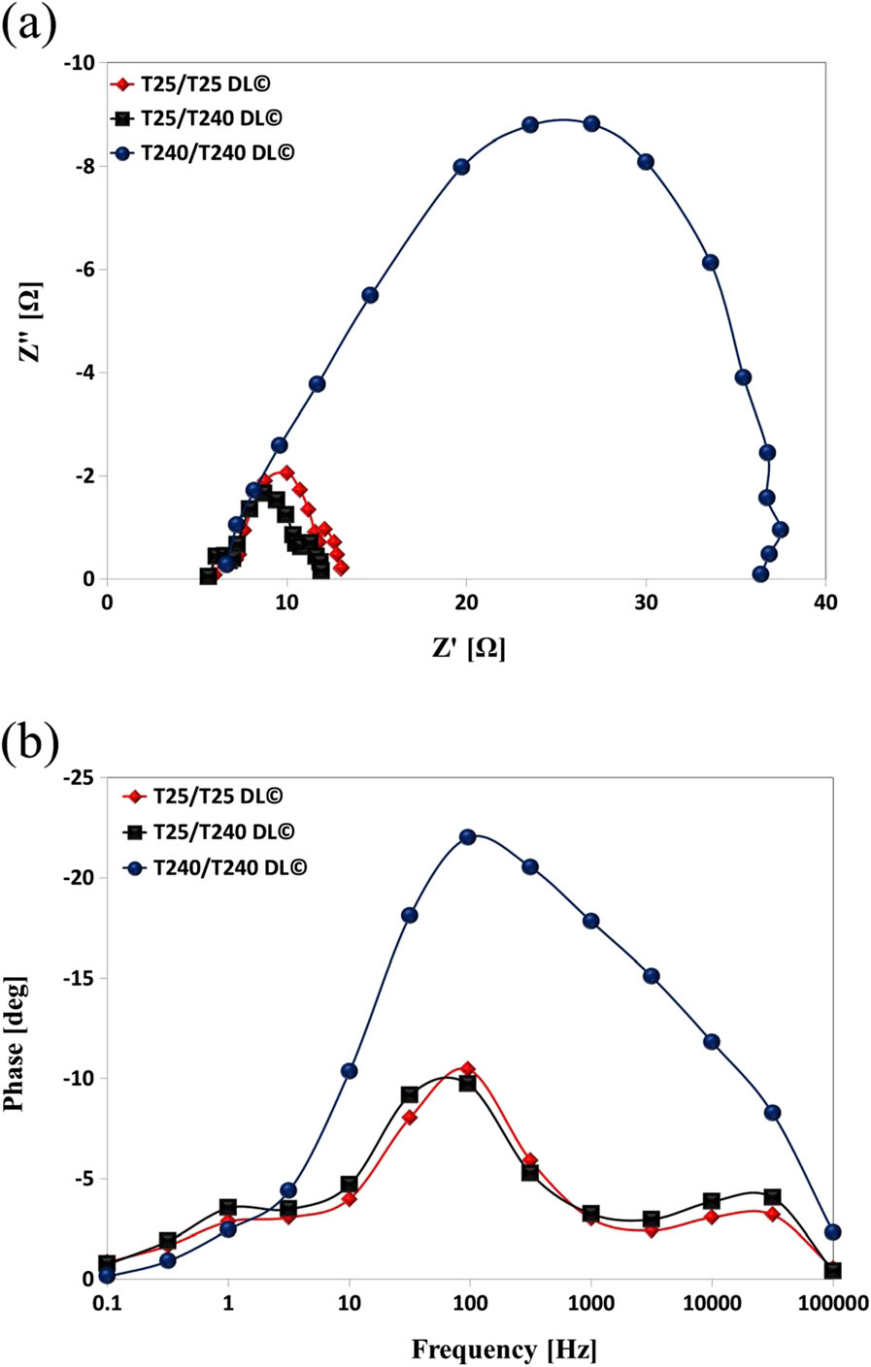
**Electrochemical impedance spectroscopy analysis of DSSCs with T25/T25, T25/T240, and T240/T240 DL.** (**a**) Nyquist plots and (**b**) Bode plots of T25/T25-DL-, T25/T240-DL-, and T240/T240-DL-based DSSCs with condenser lens-based solar concentrator.

Table 2 and Figures 3 and 4 provide further details on the photovoltaic performance of three different types of DSSCs with T25/T25, T25/T240, and T240/T240 DL. With the synergistic effect of the presence of the light-scattering layer in the photoelectrodes of DSSCs and the adoption of maximized light concentration (i.e., 3.72 Suns) in this study, T25/T240-DL©-DSSCs generated the *I*_sc_ of 11.92 mA at 0.36 cm^2^, which is comparable with the *I*_sc_ of 12.12 mA at 0.36 cm^2^ generated by T25/T25-DL©-DSSCs. However, the resulting PCE of T25/T240-DL©-DSSCs was approximately 4.11%, which is larger than approximately 3.84% of T25/T25-DL©-DSSCs. This is because the application of the light-scattering layer (T240) on top of the dye-absorbing layer (T25) (i.e., T25/T240 DL) increases light retention in the photoelectrodes of DSSCs; consequently, a considerably larger number of photogenerated electrons are injected into the TiO_2_ layer, resulting in relatively high photocurrent. Also, the adoption of T25/T240 DL© increased the resulting *V*_oc_ of 0.74 V, which is 6% increase compared to the *V*_oc_ of 0.70 V made by T25/T25 DL©. Furthermore, the increase in photogenerated electrons appears to slightly lower the recombination (*R*_rec_) and transport resistances (*R*_t_), and simultaneously increase the electron lifetime (*τ*_e_) due to increase in the diffusion coefficient of electrons. This result suggests that trapping and detrapping of electrons in TiO_2_ layers occurs at shallow levels under very high light intensity, and therefore, the electron transfer rate in the multi-layered DSSCs is considerably greater than that in the reference single-layered DSSCs.

**Table 2:** Summary of photovoltaic characteristics of DSSCs with T25/T25 DL, T25/T240 DL and T240/T240 DL

**Type**	***I***_**SC **_**(mA)**	***V***_**OC **_**(V)**	**FF**	**PCE (%)**	***R***_**rec **_**(Ω)**	***R***_**t **_**(Ω)**	***τ***_**e **_**(ms)**
T25/T25 DL©	12.12	0.70	0.61	3.84	5	5	2.0
T25/T240 DL©	11.92	0.74	0.62	4.11	3	2	3.1
T240/T240 DL©	2.21	0.77	0.47	0.60	25	12	1.3

The photoelectrodes of DSSCs with condenser lens-based solar concentrator was under the light concentration of approximately 3.72 Suns condition; T25 SL, 25-nm-sized TiO_2_ NP-accumulated photoactive layer; T25/T240 DL, 240-nm-sized TiO_2_ NP-based light-scattering layer applied on 25-nm-sized TiO_2_ NP layer; © indicates use of condenser lens-based solar concentrator; *R*_rec_, recombination resistance; *R*_t_, transport resistance; and *τ*_e_, electron lifetime.

**Figure 5:**
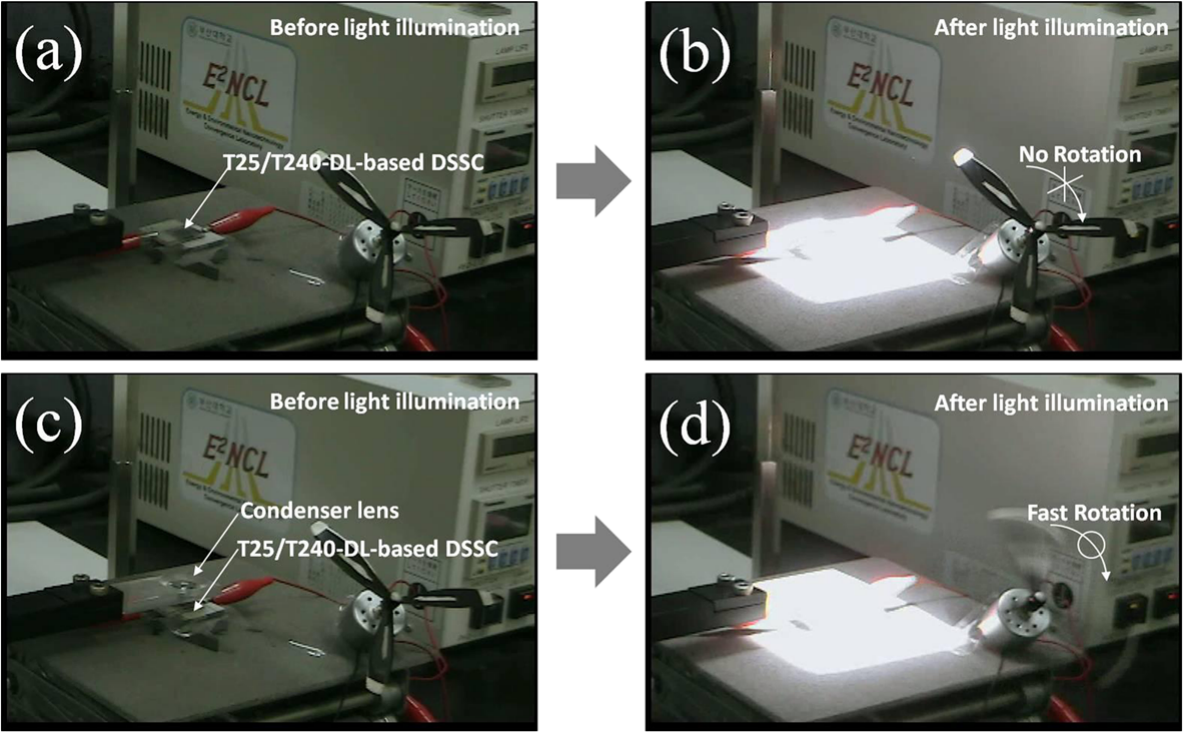
**Demonstration of high power output from solar concentrator-assisted T25/T240-DL-based DSSC.** Photographs of a propeller installed on an electric motor powered by a T25/T240-DL-based DSSC without condenser lens-based solar concentrator (**a**) before and (**b**) after light illumination (Here, the propeller did not rotate after light illumination). Photographs of a propeller installed on an electric motor powered by a T25/T240-DL-based DSSC with condenser lens-based solar concentrator (**c**) before and (**d**) after light illumination (Here, the propeller rotated very fast after light illumination).

In order to qualitatively verify the increase of power output by using the T25/T240-DL©-based DSSCs, we tried to operate a small propeller installed on an electric motor (rated voltage = 0.6 V, rated current = 12 mA, Jinlong Machinery & Electronics Co., Zhejiang, China) powered by the T25/T240-DL-based DSSC with or without condenser lens-based solar concentrator. Figure 5a, b shows that the electric motor did not operate by the T25/T240-DL-based DSSC without using condenser lens-based solar concentrator under the light illumination because the power output was not sufficient. However, after installing the light concentrator on top of the T25/T240-DL-based DSSC, the electric motor operated very fast under light illumination as shown in Figure 5c, d, suggesting that the T25/T240-DL©-based DSSC sufficiently generated power output to operate the given electric motor. This realizes a potential concept for a solar cell module composed of an optimized solar concentrator and a DSSC, which enables to operate portable electric devices with relatively high power input.

## Conclusions

In this study, we obtained the optimized intensity and focal area of incident light in a simple condenser lens-based solar concentrator by adjusting the focal length of light pathways for a reference DSSC with a T25 SL. Further, we verified the role of a T240-accumulated layer applied on top of the T25-accumulated dye-absorbing layer to serve as a strong light-scattering medium. Furthermore, the light-scattering capacity of the T240 layer in the photoelectrodes of DSSCs was found to be enhanced upon precisely concentrating the incident light with the assistance of the condenser lens-based solar concentrator. On comparison of the photovoltaic performance of the T25/T25-DL-, T25/T240-DL-, and T240/T240-DL-based DSSCs with and without the use of concentrated incident light, the optimized photoelectrode structures exhibited both enhanced dye-absorbing properties (T25) and light-scattering properties (T240) under highly concentrated light irradiation, resulting in increased values of *I*_sc_ and PCE. The presence of the light-scattering layer in the photoelectrodes of DSSCs and the use of the condenser lens system to concentrate the irradiated light can synergistically enhance the inherent photovoltaic performance of DSSCs.

## Competing interests

The authors declare that they have no competing interests.

## Authors’ contributions

KJM, SWL, and YHL contributed equally to this work as first co-authors. KJM, SWL, and YHL fabricated TiO_2_ pastes, assembled various DSSCs, and made photovoltaic performance measurement. JYA participated in the SEM measurements. SJL and DWL participated in the design and manufacture of condenser lens-based solar concentrator. SHK provided guidance to KJM, SWL, YHL, JYA, and SJL as a supervisor and designed most of this research project. All authors read and approve the final manuscript.
